# Dekyphosis operation combined with limited osteotomy to treat the symptomatic adult tethered cord syndrome with complicated malformations: A case report

**DOI:** 10.1097/MD.0000000000033600

**Published:** 2023-04-28

**Authors:** Liang Jiao, Xiao Yang, Shuang Wang, Jun-Xiong Ma, Liang Zheng, Hong Wang, Hai-Long Yu, Yu Chen

**Affiliations:** a Department of Orthopedics, General Hospital of Northern Theater Command, Shenhe District, Shenyang, China; b Department of Anesthesiology, The Air Force Hospital of Northern Theater PLA, Dadong District, Shenyang, China.

**Keywords:** kyphosis deformity, posterior vertebral column subtraction osteotomy, split cord malformation, tethered cord syndrome, thoracic disc herniation

## Abstract

**Rationale:**

Tethered cord syndrome (TCS) represents a spectrum of neurological symptoms that are caused by constant or intermittent axial traction of the terminal cone of the spinal cord due to abnormal positioning. It is uncommon for abnormal structures of TCS to be accompanied by split cord malformation, thoracic spinal stenosis, and other spinal cord diseases.

**Patient concerns:**

A 45-year-old male patient visited our hospital due to severe lower back pain, extensive left lower limb muscle weakness, and intermittent claudication.

**Diagnoses:**

TCS combined with stenosis of the thoracic canal, split cord malformation, and kyphosis deformity.

**Interventions:**

The patient underwent Dekyphosis operation combined with limited osteotomy symptoms.

**Outcomes:**

The patient felt the right lower limb improved after surgery. At 4-month follow-up, a radiological examination showed adequate decompression of the spinal cord and a good internal fixation position. Overall, the patient’s clinical symptoms significantly improved.

**Conclusion:**

This is a rare case of TCS combined with thoracic disc herniation and bony mediastinum. A more conservative invasive surgical approach was elected and markedly improved the patient’s symptoms. Additional clinical cases are needed to confirm the stability and feasibility of this surgical approach.

## 1. Introduction

Tethered cord syndrome (TCS) involves a series of ischemic pathological changes caused by the incommensurate shortening of the spinal cord compared with the bony structure of the spine.^[1]^ These changes include the lower position of the conus medullaris, but also the thickening of the fibrous adhesion and the hyperplasia of the fatty mass around the dura could be observed.^[2]^ The symptoms include the feeling and motion abnormalities of the lower limbs and vary according to the extent of the unnormal anatomical structure. Also, in some patients, urinary function could be influenced.^[3,4]^ Generally, the symptoms manifest during childhood, and the patients can be diagnosed and treated timely. Detethering surgery is a standard procedure for TCS in most children and some adults.^[5]^ The efficacy and prognosis of all kinds of surgeries, including detethering, are satisfactory when they are performed during childhood. However, surgery to treat symptomatic TCS during adulthood can be challenging and complicated.^[6,7]^ In these cases, several kinds of malformations might also be diagnosed and should be treated in the meantime. Although it is unknown whether these malformations are the consequences of symptomatic adult TCS or the correction of the body to the unnormal anatomic structure, they should be corrected to some degree.^[8,9]^ Many types of surgery have been performed in order to treat adult TCS, but it is still far from a consensus on how to treat these complicated cases, and lots of observations and research should be performed. In the current case, an individualized surgery plan was presented to treat a combination of the symptomatic TCS combined with split cord malformation (SCM), thoracic spinal stenosis, and kyphosis. For treating the patient, the operations of dekyphosis and limited osteotomy were performed in order to correct the kyphosis and relieve the compression. By this operation, the symptoms of the patient were relieved obviously.

## 2. Ethical statement and consent

The Institutional Review Board and Ethics Committee of the General Hospital of Northern Theater Command approved this investigation. Written informed consent was obtained from the patient for this case report to be published (including images, case history, and data).

## 3. Case presentation

### 3.1. Patient information

A 45-year-old male with no previous diagnosis of TCS presented severe numbness and pain in both lower extremities accompanied by difficulty in walking. One year before presenting at our hospital, symptoms developed in both of his lower limbs without obvious inducement. No systematic treatments were applied. One month prior to revisiting the hospital, the patient’s symptoms had worsened to the point that weakness in both lower limbs compromised his ability to walk (Table 1). Despite application of a variety of conservative treatments, none of the patient’s symptoms improved.

**Table 1:** Timeline.

Jun 01, 2021	Numbness manifests in both lower limbs and does not receive special treatment.
May 30, 2022	Patient’s symptoms are aggravated.
Jun 15, 2022	MRI and CT examination show extrusion of the thoracic vertebral disc (T11–12), stenosis of the thoracic canal, posterior convex deformity of the thoracic spine, a bony structure splitting the cord into 2 hemi cords, and abnormal fixation of the spinal cord.
Jun 30, 2022	Admittance to hospital
Thoracic CT, chest X-ray, and tests of blood and urine were performed	
July 05, 2022	A limited posterior vertebral column subtraction osteotomy was performed to correct the deformity and shorten the spine. Discectomy of T11–12 and a fixing operation from T8–L2 was also performed.
July 09, 2022	DR and MRI were performed post-surgery. Patient’s symptoms were relieved.
July 15, 2022	Patient discharged
November 07, 2022	Patient follow-up

CT = computed tomography, DR = digital radiography, MRI = magnetic resonance imaging.

### 3.2. Physical exam

Examination showed weakness of the right extensor hallucis longus (Medical Research Council 3/5). On the left side, the extensor digitorum longus, flexor hallucis longus, and flexor digitorum longus were also reduced to medical research council 3/5. Severe weakness of the right quadriceps femoris was observed with muscle strength of grade 0. Sensory of the right lower extremity had declined, especially in the lateral of the crus and at the bottom of the foot. The knee tendon reflexes were hyperactive on both sides, and Babinski sign was positive on both sides. Other tendon reflexes and pathological signs were normal.

### 3.3. Diagnostic assessment and therapeutic intervention

**Figure 1.**
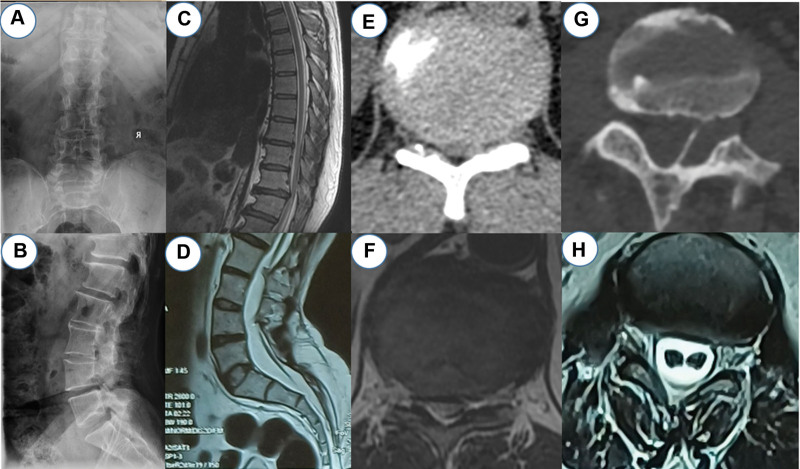
Imaging examinations performed prior to surgery. Anteroposterior (A) and lateral plain (B) radiographs of the lumbar spine. T2-weighted sagittal MRI of the lumbar spine showed an abnormal herniated disc at the T11-12 level and a low conus medullaris at the L4 level. (E and F) CT and axial MRI of the thoracic spine confirmed thoracic disc herniation and spinal stenosis at T11/T12. (G and H) CT and axial MRI of the lumbar spine show the bony structure splitting the cord into 2 hemi cords. CT = computed tomography, MRI = magnetic resonance imaging.

The computerized tomography scan was performed to specify the origin of the progressive loss of sensation and motor functions that manifested. Thoracic disc herniation and spinal stenosis at the T11/T12 level were detected. Magnetic resonance imaging further showed an abnormal herniated disc at the T11/12 level and a bony type 1 split cord malformation SCM, also, the conus ended at approximately the L4 level (Fig. 1).

After detailed preparation and discussion, a posterior operation was performed. A laminectomy decompression of T9 to L2 was performed, with a *discectomy* of T11 to 12 and autogenous bone grafted into the intervertebral space to realize interbody fusion. Prior to the fusion procedure, the limited osteotomy was performed in the facet joint on both sides. By these means, the kyphosis of the patient was corrected and the tension of the spinal cord was released. Then the fixation from T8 to L2 was achieved using a pedicle screw-rod system.

### 3.4. Follow-up and outcome

**Figure 2.**
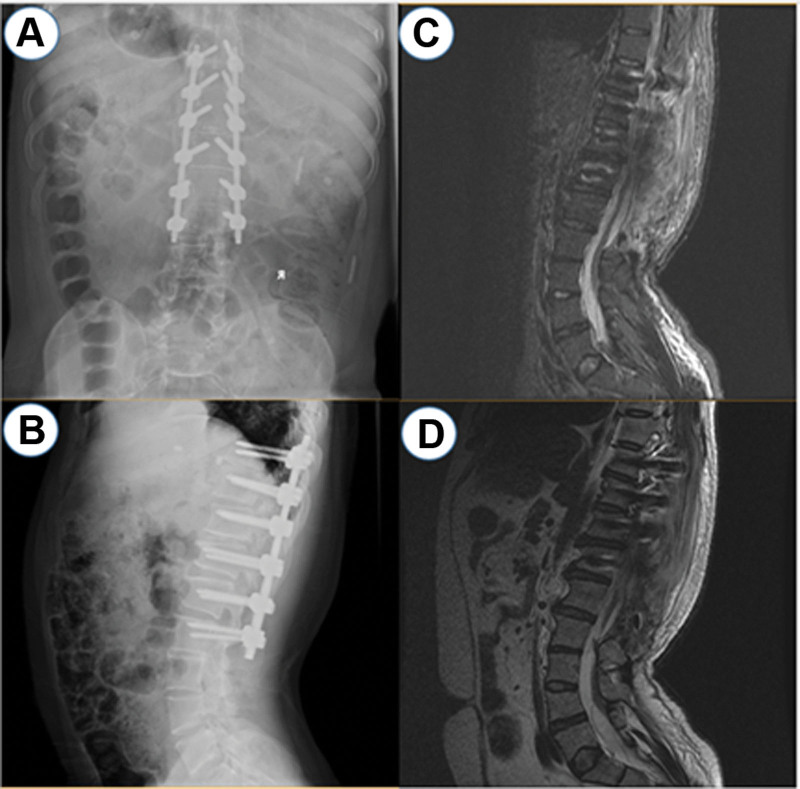
Anteroposterior (A) and lateral (B) plain radiographs of the lumbar spine obtained post-surgery. An improved sagittal profile is observed. T2-weighted sagittal MRI of the lumbar spine also taken post-surgery at the T9–T12 level (C) and of the low conus medullaris at the L4 level (D). MRI = magnetic resonance imaging.

**Figure 3.**
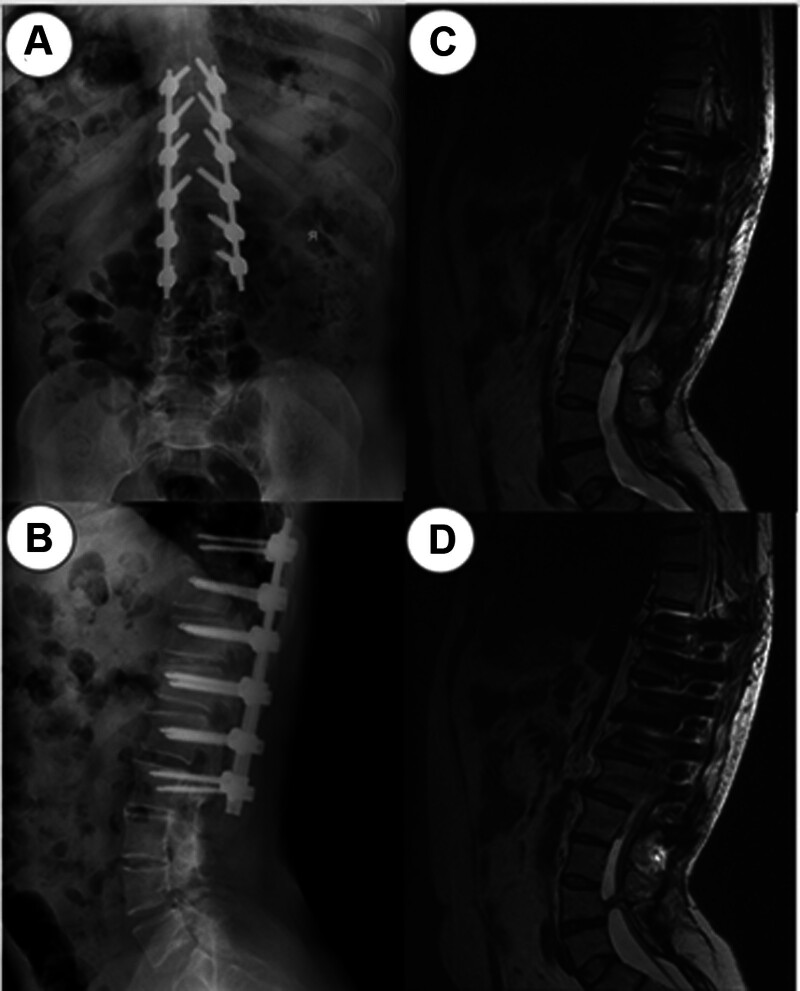
Anteroposterior (A) and lateral (B) plain radiographs of the lumbar spine at 4 months after surgery. Curvature of the spine was well maintained and no re-deformation occurred after correction. (C and D) T2-weighted sagittal MRI of the lumbar spine shows a good internal fixation position. MRI = magnetic resonance imaging.

Exploration showed that tension of the dural sac decreased, and fluoroscopy confirmed that the degree of kyphosis was significantly improved after surgery. There were also no complications during or after surgery. Good lower limb activity and muscle strength were observed, and the patient’s subjective symptoms were significantly relieved. The patient’s spinal deformity was also improved according to imaging (Fig. 2). After 4 months, the patient returned to the outpatient clinic of our hospital for reexamination. Both digital radiography and magnetic resonance imaging results showed that the internal fixation was in a good position, and the patient’s self-reported symptoms were significantly improved (Fig. 3).

## 4. Discussion

Tethered cord syndrome is usually described as degenerating due to the abnormal fixation of the conus medullaris to the lower end of the spine for some reason, resulting in ischemia and hypoxia of the spinal cord.^[10]^ Clinically, some patients will have symptoms of nerve damage such as lower limb weakness, numbness, small limbs, foot deformities, defecation, and urination disorders.^[11]^ TCS mostly occurs in childhood.^[12]^ Although adult patients with primary TCS have spinal cord traction factors, during development, the spinal cord function and traction factors reach a critical balance, and there are often no obvious symptoms in the early stage.^[13]^ However, this balance is very fragile and often broken under inducements such as trauma, and the corresponding clinical symptoms will appear at that time.^[14]^ At present, surgical treatment is still the first choice for symptomatic adult patients.^[7,15]^ Classical Untethering Surgery has been the gold standard to date.^[15–17]^ The tension of the spinal cord was reduced by releasing the traction and fixation of the spinal cord through the thickening of the filum terminale. Scoliosis correction surgery was performed 3 to 6 months later.^[12,18]^ Although this method has achieved some therapeutic effects, a large number of studies have shown that tether release is generally better for children with a tethered spinal cord, but for adult patients, this treatment method has a high incidence of complications, a high proportion of incomplete tether release, and a high rate of re-tethering.^[19–21]^ In addition, secondary surgery and secondary anesthesia are required, increasing the patient’s pain and the risk of surgery.^[22]^ In order to seek a safer and better-prognosis surgical scheme. Kokubun made the initial suggestion that posterior vertebral column subtraction osteotomy be used as the primary treatment for individuals with lower TCS of the conus medullosus in 1995.^[23,24]^ A large number of clinical trials have confirmed that posterior vertebral column subtraction osteotomy has a good effect on symptomatic adult patients with a tethered cord.^[25–31]^

SCM, which accounts for 5% of cases of spinal dysraphism, is characterized by a longitudinal division of the spinal cord into 2 distinct hemi-spinal cords.^[32]^ Pang and colleagues classified type I SCM (2 hemicords, each in its own dural tube, separated by a dural-sheathed osseous or cartilaginous medial septum) and type II SCM (2 hemicords in a single dural tube divided by a nonrigid, fibrous septum).^[33]^ TCS is the most common complication of SCMs in adults.^[34,35]^ Common causes of symptoms in tethered cord patients with SCM include fixation of the hemi-spinal cord by a bony (type I SCM) or fibrous (type II SCM) septum.^[36,37]^

For patients with a tethered spinal cord combined with a split spinal cord, the tethered spinal cord is usually released by resection of the bony mediastinum.^[38–40]^ However, considering the complicated situation such as thoracic disc herniation and kyphosis in this case, in order to prevent the possibility of a large operation scope, a long operation time, excessive bleeding, etc. We propose a dekyphotic correction combined with a posterior osteotomy. Our goal was to address the patient’s pain and weakness while minimizing trauma. Due to the instability and compression caused by kyphosis, immobilization is largely required to obtain stability. In addition, compared with tethered cord release alone, dekyphosis combined with limited osteotomy is a feasible method to avoid intensive manipulation of neural structures and reduce neurological complications.^[41]^ This surgery reduces the traction on the dural sac and prevents further aggravation of tethered cord symptoms. At the same time, the scope of surgery is smaller, and there is no need for secondary surgery to release the tethered cord. The risk of anesthesia and surgery is reduced. Postoperative imaging showed that the tension of the spinal cord and the degree of kyphosis were improved to a certain extent by dekyphosis combined with limited osteotomy. In this case, the walking function gradually recovered after the operation, and the pain and numbness were greatly relieved. At the same time, there were no expected complications such as cerebrospinal fluid leakage or lower limb symptoms aggravation.

## 5. Conclusion

There are multiple surgical options for treating symptomatic adult patients with TCS. The treatment of these patients with a bony mediastinum and split cord could be more complicated with greater challenges. Therefore, limited procedures may be more successful in the absence of detethering or vertebral column shortening surgeries. Use of dekyphosis operation combined with limited osteotomy in the present case demonstrates the advantages and success of this type of approach. However, additional studies are needed to determine the durability of dekyphosis operation combined with limited osteotomy for the treatment of adult TSC when additional conditions such as bony mediastinum, dichotomous spinal cord, thoracic disc herniation with kyphosis, and split spinal cord are present. The present case report demonstrates that limited operations can potentially achieve good results if they are carefully designed and individualized to the patient.

## Acknowledgements

We thank *Medjaden* Inc. for scientific editing of this manuscript.

## Author contributions

**Conceptualization:** Yu Chen.

**Writing – original draft:** Liang Jiao, Xiao Yang, Shuang Wang.

**Writing – review & editing:** Liang Jiao, Shuang Wang, Jun-Xiong Ma, Liang Zheng, Hong Wang, Yu Chen, Hai-Long Yu.
